# Preclinical evaluation of a tool for insertion force measurements in cochlear implant surgery

**DOI:** 10.1007/s11548-023-02975-2

**Published:** 2023-06-13

**Authors:** Georg Böttcher-Rebmann, Viktor Schell, M. Geraldine Zuniga, Rolf Salcher, Thomas Lenarz, Thomas S. Rau

**Affiliations:** Department of Otolaryngology and Cluster of Excellence EXC 2177/1 “Hearing4all”, Hannover Medical School, Carl-Neuberg-Str. 1, 30625 Hannover, Germany

**Keywords:** Cochlear implant, Insertion forces, Intraoperative insertion force measurement, Manual insertion tool

## Abstract

**Purpose:**

Trauma that may be inflicted to the inner ear (cochlea) during the insertion of an electrode array (EA) in cochlear implant (CI) surgery can significantly decrease the hearing outcome of patients with residual hearing. Interaction forces between the EA and the cochlea are a promising indicator for the likelihood of intracochlear trauma. However, insertion forces have only been measured in laboratory setups. We recently developed a tool to measure the insertion force during CI surgery. Here, we present the first *ex vivo* evaluation of our tool with a focus on usability in the standard surgical workflow.

**Methods:**

Two CI surgeons inserted commercially available EAs into three temporal bone specimens. The insertion force and the orientation of the tool were recorded together with camera footage. The surgeons answered a questionnaire after each insertion to evaluate the surgical workflow with respect to CI surgery.

**Results:**

The EA insertion using our tool was rated successful in all 18 trials. The surgical workflow was evaluated to be equivalent to standard CI surgery. Minor handling challenges can be overcome through surgeon training. The peak insertion forces were 62.4 mN ± 26.7 mN on average. Peak forces significantly correlated to the final electrode insertion depth, supporting the assumption that the measured forces mainly correspond to intracochlear events and not extracochlear friction. Gravity-induced forces of up to 28.8 mN were removed from the signal, illustrating the importance of the compensation of such forces in manual surgery.

**Conclusion:**

The results show that the tool is ready for intraoperative use. In vivo insertion force data will improve the interpretability of experimental results in laboratory settings. The implementation of live insertion force feedback to surgeons could further improve residual hearing preservation.

**Supplementary Information:**

The online version contains supplementary material available at 10.1007/s11548-023-02975-2.

## Introduction

A cochlear implant (CI) is a neuroprosthesis used to treat sensorineural hearing loss. During CI surgery, an electrode array (EA) is inserted into the inner ear (cochlea), where it directly stimulates the auditory nerve. The insertion of the EA is a critical step, especially for patients with residual hearing. These patients greatly benefit from the preservation of their residual hearing, as the combination of electrical and acoustic hearing, so-called electric acoustic stimulation (EAS), facilitates superior hearing outcomes [1, 2]. During the insertion of the EA, intracochlear structures can be damaged, thus impairing residual hearing [3, 4]. As a result, modern CI surgery aims to prevent trauma by incorporating the soft-surgery approach, which is more delicate and promotes EAS [5]. A promising indicator for the likeliness of trauma infliction during the insertion are the forces exerted by the EA within the cochlea [6]. Laboratory research investigated the susceptibility of different intracochlear membranes to external forces [7, 8] as well as factors influencing the magnitude of insertion forces [9–12]. An improved understanding and measurement of insertion forces will guide future CI surgery [13]. Before patients can benefit from these results, they still need validation through data obtained during real CI surgeries. However, forces have never been measured during a real EA insertion.

We recently presented a manual insertion tool that can measure the insertion forces during standard CI surgery [14]. The design of the tool was tested and the quality of obtainable measurements evaluated in laboratory conditions, showing that the measurement of insertion forces with a manual tool is possible. The variable spatial orientation of the tool due to manual use causes gravity-induced forces that are superimposed on the force signal. To account for this, an algorithm to remove these forces using acceleration data from an inertial measurement unit (IMU) was implemented and validated.

In this work, we present an ex vivo evaluation of the tool. The main focus is evaluating the usability of the tool within the workflow of standard CI surgery. While we had performed only a preliminary surgical workflow evaluation in our previous work, a systematic evaluation can provide valuable insight and thereby prepare the successful transition into the operating room (OR). Another central aspect is the investigation of data recorded in a more realistic environment. Initially, the evaluation of the tool was performed on artificial cochlea models with an EA dummy. It is unclear how data from real EAs inserted into human cochlea specimens differs from data obtained in a test bench both in magnitude as well as in form. Moreover, the simplified artificial cochlea models might lack procedural challenges present in human specimens. The present study now focuses on the insertion of cochlear implant EAs into human temporal bone specimens with conditions closer to the intraoperative setting. Additionally, we sought to assess whether our tool is transferable to the OR and whether meaningful data can be obtained through manual measurements.

## Methods

### Experimental setup

For an adequate assessment of the usability of the tool within the surgical workflow, the experimental conditions were designed to be as close to reality as possible. Adult temporal bone (TB) specimens with previously unopened cochleae, freshly frozen postmortem and thawed immediately before the experiments, were used. While there are still differences in tissue properties compared to those in a human patient, freshly frozen TB tissue properties are more realistic than those of fixated specimens, therefore yielding a more realistic experimental environment.

**Fig. 1:**
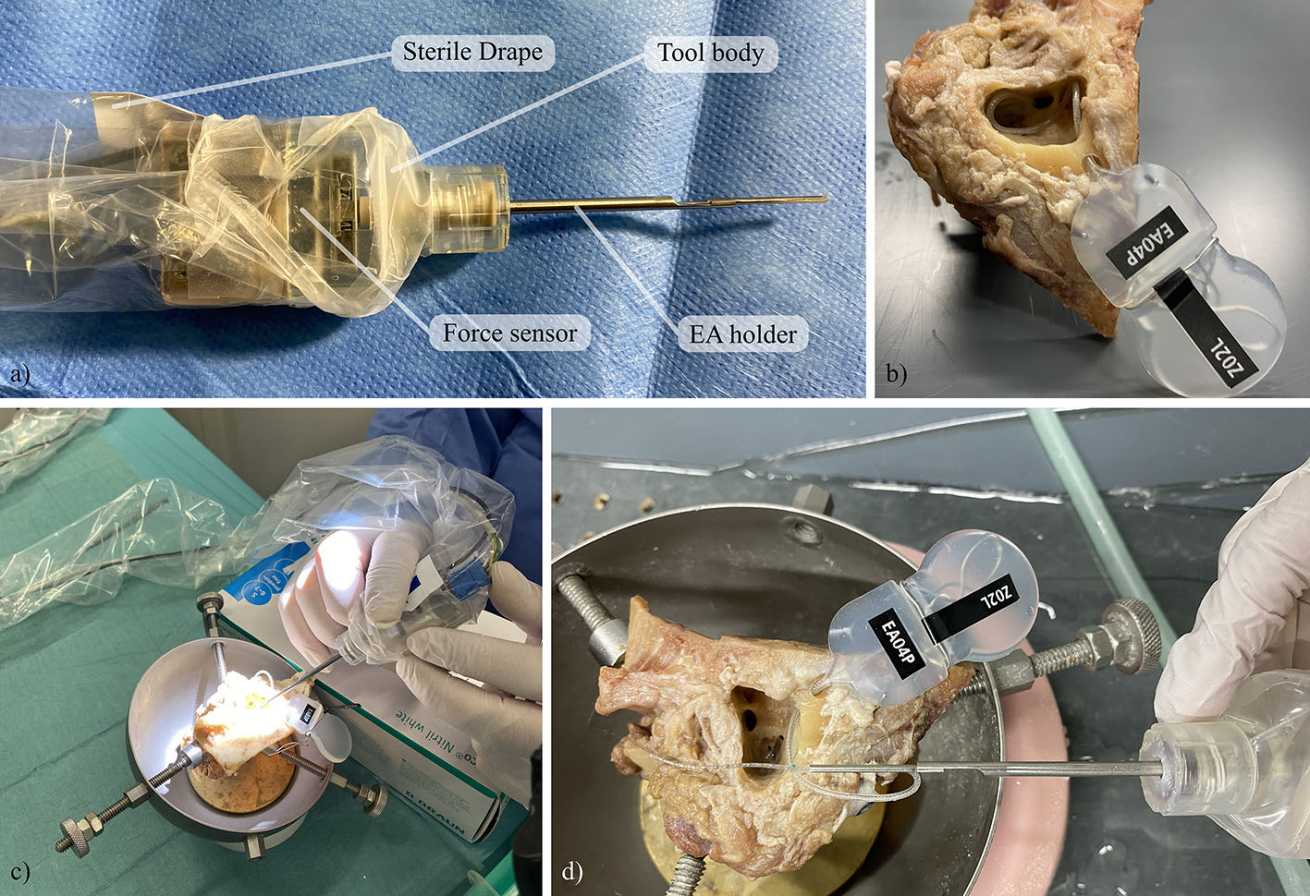
Experimental setup with prepared manual insertion tool (**a**), prepared TB specimen (**b**), insertion procedure (**c**) and TB specimen with inserted EA (**d**)

Two of the three TB specimens used were from the left and one from the right side. A surgeon prepared the specimens for the experiments by drilling a mastoidectomy and posterior tympanotomy with a round window access to the cochlea. A bone bed for the implant processor could not be drilled due to the limited size of the TB specimens and instead the processor was fixed in place with a wire (see Fig. 1b and d)). A tunnel for the EA lead was drilled from the processor position into the mastoidectomy to ensure that the length and angle of the electrode lead were comparable to standard CI practice. As usually performed in our clinic, a bone slit was added in the inferior corner of the tympanotomy to later clamp the EA in [15]. After preparation, a pre-insertion cone beam computed tomography (CBCT) scan was performed.

For the insertions, six commercially available EAs were used (*Flex24, MED-EL, Innsbruck, Austria*). The EAs included a silicone processor dummy to ensure realistic lead lengths during the insertions.

The insertion force measurement tool was prepared for surgery as described in [14]. A different EA holder was used for right and left TB specimens to ensure the EA could be easily released toward the bone slit after the insertion. A custom-made software was used to record the measured force and the spatial orientation from the tool. Additionally, a camera captured the view through the OR microscope and another camera recorded the tool and the hands of the surgeons from the side. All video and sensor data was synchronized so that events in the video would correlate to events in the sensor data [16]. The current forces were not communicated with the surgeons, as the evaluation of feedback exceeded the scope of this study.

Two CI surgeons performed insertions with the tool. Each surgeon performed a cycle of three insertions with the same EA into each TB specimen. A new EA was used for every surgeon and TB specimen, respectively. In the design of the study, changes in tissue properties in the TB specimens and mechanical properties of the EAs caused by multiple insertions were expected. As the focus of this study was on the usability evaluation and an exploration of insertion forces, the varying conditions enable a broader spectrum of insertion variations that can occur and corresponding forces that can be measured. For the same reason, no reference insertions using standard methodology were performed, as the results from such insertions would be hard to compare to the insertions with the tool due to the varying conditions. More importantly, such a reference insertion would not include force measurement and therefore a comparison with respect to forces would not be possible. A CBCT scan of the TB with the inserted EA was performed after each insertion. The experimental setup is shown in Fig. 1.

### Evaluation methods

The surgeons were asked to answer a questionnaire after each insertion in order to evaluate the insertion and to compare it to the standard surgical workflow. The first question asked whether they would assess the insertion as successful. Possible factors influencing this evaluation include the possible insertion depth, buckling of the EA during the insertion or a possible complication or prolongation of the insertion procedure. The question however aimed more at the *subjective* satisfaction of the surgeons with the insertion process rather than an objective assessment of the completeness of the insertion, which is why the specific evaluation of success was left to the surgeon. If the answer to the first question was *no*, they were asked to estimate, whether this was due to the tissue properties of the specimen or due to the use of the tool. For a more objective measure, the electrode insertion depth was also measured in the post-insertion CBCT scans. Following [17], an electrode insertion depth of 22 mm or greater, counted from the round window (RW), was regarded as complete. The next set of questions focused on the insertion and asked surgeons to evaluate procedural elements compared to the standard methodology. As the main difference is the way the EA is held, surgeons should evaluate whether the tool held the EA securely. Then they were asked to state whether their range of motion was sufficient to assess whether the use of the tool influences the manual dexterity. Similarly, surgeons were asked to rate whether the housing of the tool interfered with the visibility of critical anatomical structures. In the last two questions, the surgeons had to evaluate the complexity of the loading and offloading of the electrode array, which fundamentally differs from using standard surgical forceps or tweezers. The full questionnaire is shown in Table 1.

**Table 1:** Questionnaire answered by surgeons after each insertion

Question number	Statement	Reply options
Q1	The insertion was successful	□Yes □ No
Q1.a)	If the insertion was deemed unsuccessful, to what extent is this presumably rooted in the TB specimen and in the tool?	_____ % Specimen _____ % Tool
Q2	The tool securely holds the electrode array	When compared to conventional surgical methodology… □ Better/easier □ Equal □ Inferior without patient safety concerns □ Inferior with patient safety concerns □ No statement possible
Q3	The range of motion during insertion is sufficient	
Q4	The tool does not interfere with visibility throughout the insertion	
Q5	The electrode array could easily be loaded into the tool	□ Easy □ Moderate □ Tedious □ Hard □ No statement possible
Q6	The electrode array could easily be offloaded from the tool	

As the electrode insertion depth was not continuously measured during the insertions, the forces recorded with the tool were evaluated by computing the maximum force. The data on the orientation of the tool was used to calculate adjustments to the trajectory performed by the surgeons during the insertion as these adjustments also influence the gravity-induced forces disturbing the measurement. The orientation data is not registered to the specimens, therefore no spatial directions but only the absolute adjustment with respect to the start orientation is considered. The magnitude of these forces was calculated as well to assess the necessity of the compensation algorithm implemented with the tool. Additionally, the forces recorded with the tool were evaluated for unusual peaks or patterns. If present, the synchronous video was used to identify a cause.

## Results

In the questionnaire, the surgeons evaluated 83% (*n* = 15) of the trials as successful. For all unsuccessful trials (*n* = 3), the surgeons stated in question 1a) that this was due to the tissue properties of the TB specimen and not due to handling issues with the tool. The EA was securely held by the tool in all trials according to the surgeons. In one trial, the surgeon preferred this to the conventional method, as they were able to concentrate on their hand motion and not holding the EA. The range of motion allowed by the tool during the trials was rated to be equivalent to standard methodology in all trials. The tool did not interfere with the visibility of critical anatomical structures in all trials.

The first surgeon stated that loading the EA into the tool was easy in all trials. The second surgeon evaluated the loading of the electrode to be moderately easy in their first and third trial (11%). In both cases, this did not affect the electrode insertion depth.

The offloading procedure was also evaluated to be easy in most trials. In one trial (6%) it was rated as moderately easy as the way a forceps was used for offloading was slightly changed, complicating the procedure. Offloading was described as tedious in two trials (11%). In the first, the EA was clamped too close to the tip, requiring the surgeon to insert the EA holder through the facial recess. In addition, the tool was rotated during the insertion, which is why the opening of the EA holder did not face the bone slit. In the following trials, the surgeon made sure that the point where the EA was held and the tool rotation were correct which prevented such a situation. In the second trial with offloading rated tedious, the surgeon had to cross their hands while performing the insertion with the right hand but had to offload the EA to the right with the left hand. It was later stated that this could have been prevented by either using the left hand for the insertion or swapping hands for offloading. In contrast to both aforementioned trials, the offloading issues might have moderately affected the electrode insertion depth in this trial, as this was a trial where the last contact of the EA was at the height of the RW.

**Fig. 2:**
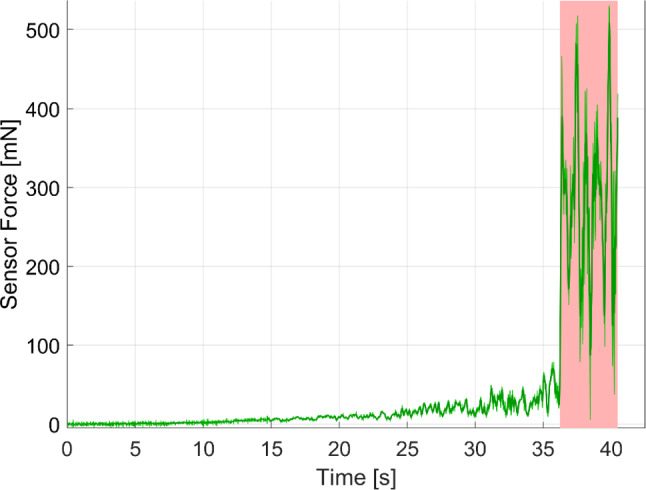
Forces including bone contact (marked red) for trial 15

Forces and rotational movements during the insertion were reliably recorded. Although the measurements were successful, three of the 18 trials included strong interference forces. These were caused by contact between the EA holder and bone or other tissue. Surgeons either accidentally contacted bone during the insertion or rested the EA holder on the bone to gain more stability. As these contact forces are at least an order of magnitude higher than the expected insertion forces and cannot be compensated for, they are excluded from the measurement. Time periods during which bone contact occurred were identified from the recorded videos and confirmed in the force data. In the three trials these events were present for 17.1%, 7.6% and 10.6% of the time, respectively. Nonetheless, the forces recorded after a bone contact event are still valid, as the measurement is immediately clear from the undesired external force and the sensor therefore only measures the forces transmitted through the EA. An illustrative force profile for a trial with bone contact is shown in Fig. 2.

**Fig. 3:**
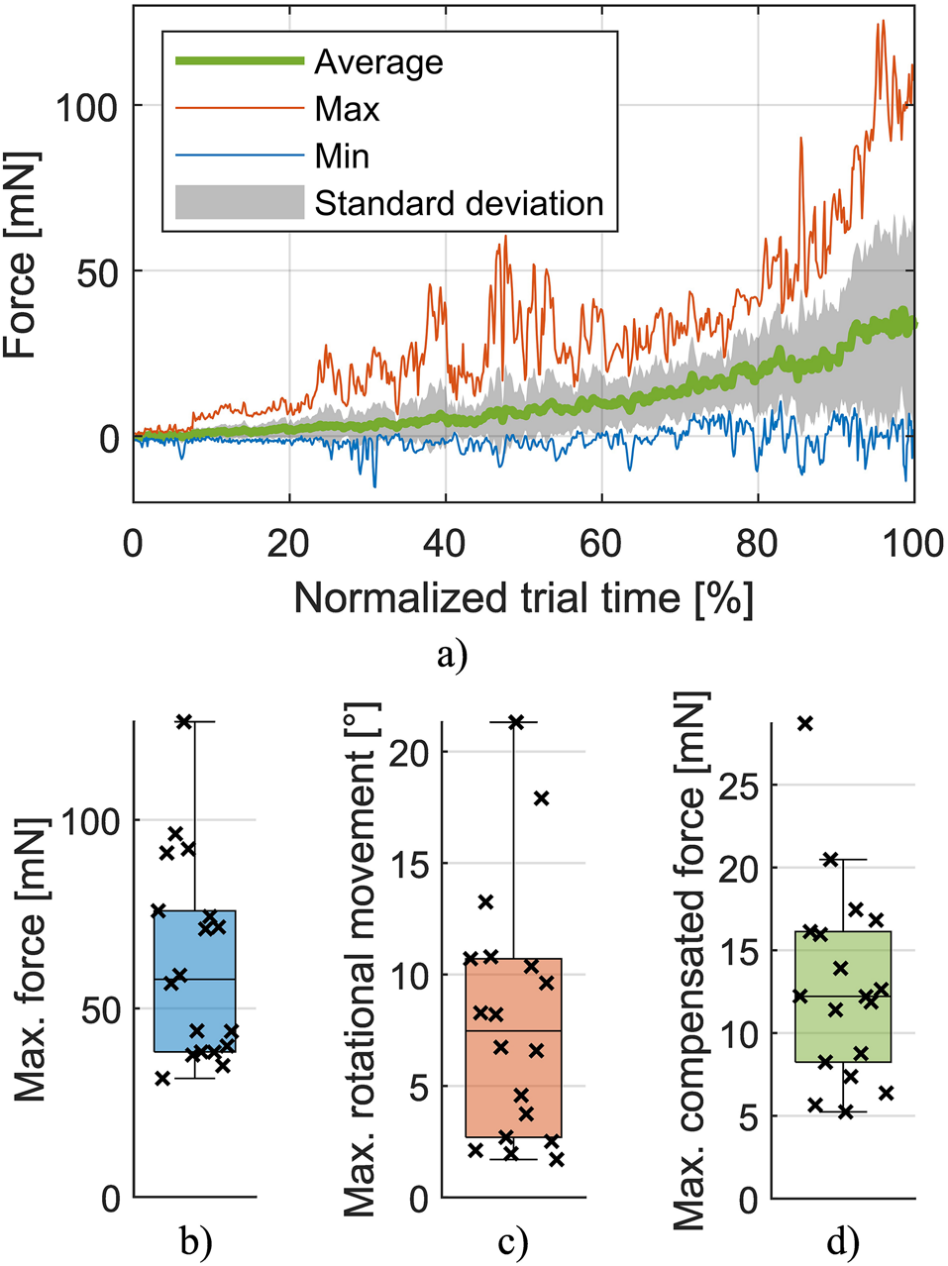
Summary of measured data from the trials. Average force profile with standard deviation, minimum and maximum values determined from all trials with respect to the normalized trial time (**a**). Largest forces when omitting bone contact (**b**), largest rotational movement performed by the surgeon (**c**), largest gravity-induced forces compensated from the force signal (**d**)

The measurements for the respective trials showed some variation. The duration of the insertion ranged between 14 and 78 s with a mean duration of 38.3 s ± 18.8 s over all trials. For a better overview of the distribution of the forces, they are shown in Fig. 3a with normalized trial time. When removing periods with bone contact from the trials, the largest force (usually measured at the end of the insertion) was on average 62.4 mN ± 26.7 mN with values ranging from 31.4 mN to 126.0 mN. The distribution of these maximum forces is displayed in Fig. 3b.

The rotational movements the surgeons performed with the tool also varied between the trials. Some insertions were almost linear with an angular adjustment of just 1.7° with respect to the initial trajectory, while others included large rotational movements of up to 21.3° (see Fig. 3c). Particularly with larger adjustments, the data was made more reliable through the compensation of gravity-induced forces. These would have disturbed the measurements with magnitudes of up to 28.7 mN and an average of 12.9 mN ± 5.9 mN as shown in Fig. 3d.

**Fig. 4:**
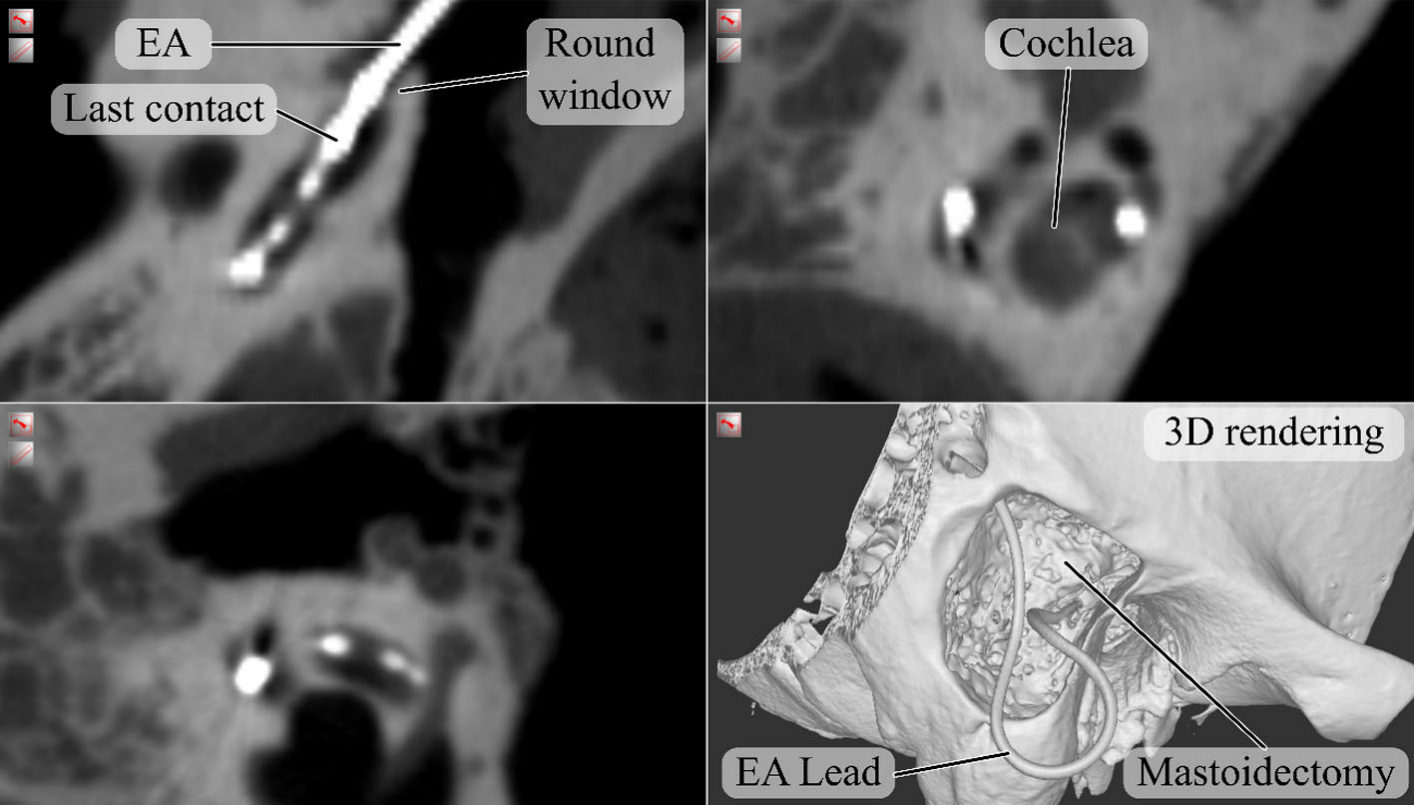
Typical CBCT scan of a TB specimen with inserted electrode and highlighted key structures

**Fig. 5:**
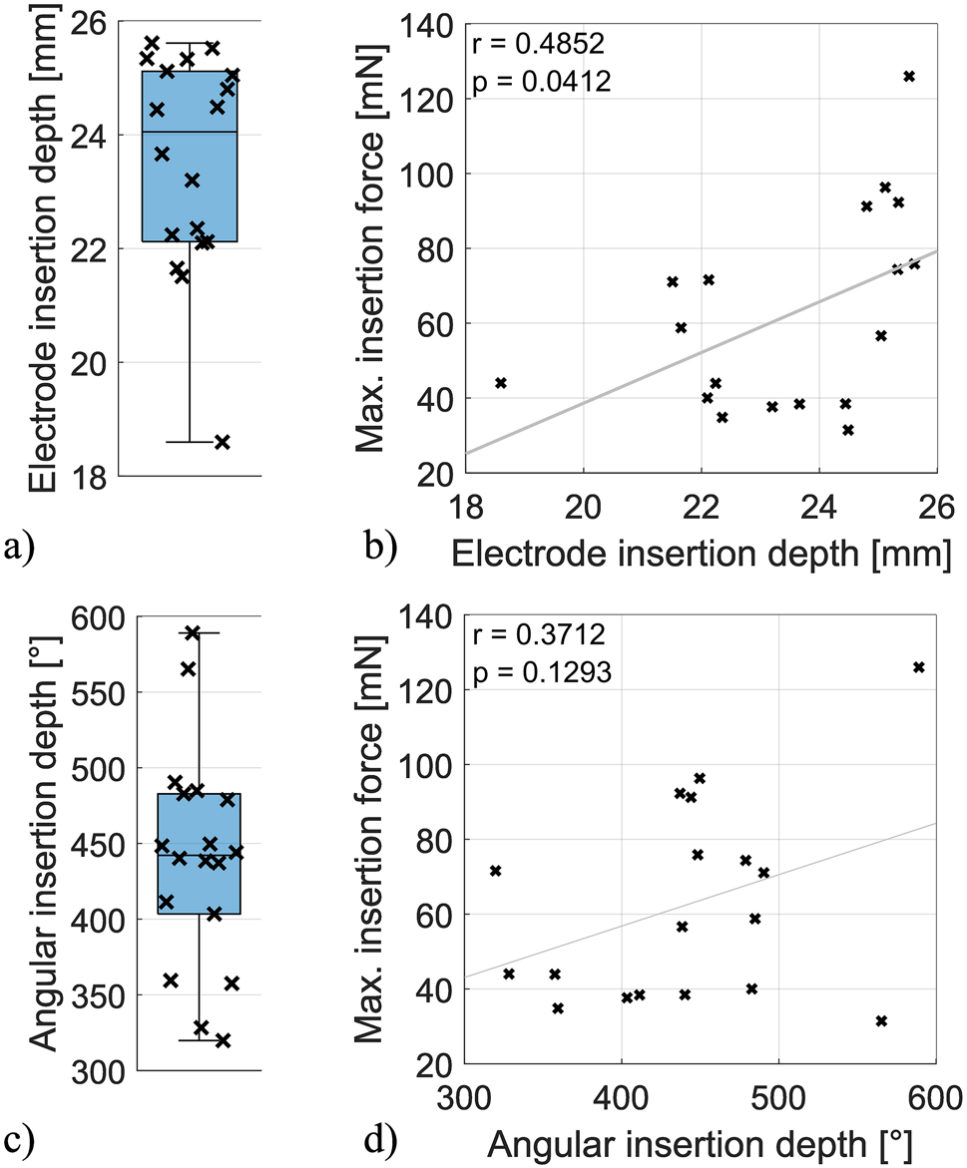
Correlation of insertion depth measurements with max. forces (omitting periods with contact between the EA holder and bone). Least squares trend lines highlighting the correlation. Measured electrode insertion depth (**a**) and correlation with the max. insertion forces (**b**) as well as measured angular insertion depth (**c**) and correlation with max. insertion forces (**d**)

The analysis of the CBCT scans (see Fig. 4) showed that all EAs were inserted with minor difference in electrode insertion depth in all trials. In the very first insertion into one TB, the EA could only be inserted up to the 11^th^ contact at the RW due to larger intracochlear resistance. This insertion was also rated unsuccessful by the surgeon. For all following trials, deeper insertions were possible with the last contact just at the RW or deeper. The average electrode insertion depth was 23.5 mm ± 1.9 mm (see Fig. 5a) and the average angular insertion depth was 440.5° ± 72.1° (see Fig. 5c). After the insertions, the EA leads were clamped firmly in place within the bone slit in all trials. Upon closer inspection of the deeper electrode contacts, a tip fold-over was detected in the fifth trial. This was not noticed during the insertion.

To assess whether the variable insertion depth was reflected in the peak insertion force, a correlation analysis was performed for the electrode insertion depth and the angular insertion depth. When removing bone contact, the force maxima significantly show a moderate linear correlation with the electrode insertion depth (*r* = 0.4852, *p* = 0.0412). No significant correlation with the angular insertion depth was found (*r* = 0.3603, *p* = 0.1419). Correlation graphs are shown in Fig. 5b, d, respectively.

## Discussion

The insertion force measurement tool was successfully used in a realistic environment. Surgeons were able to perform CI EA insertions and evaluated the handling to be equivalent to the standard surgical approach. While in some cases the EA was not fully inserted, this was likely caused by the limiting tissue properties of cadaver specimens, according to the surgeons. The loading and offloading of the EA using our tool, which poses the only real procedural difference with respect to standard surgery, was mostly easy for the surgeons. In the singular cases where they found it more challenging, they stated that this was due to experimental changes in the method they used. A thorough training would aid surgeons in finding an approach that suits their technique with which they would feel comfortable. Apart from these factors, the intraoperative workflow developed for the tool was successfully implemented in all trials. This included tool calibration, sterile assembly, handling through a sterile drape and disassembly. While the design of the EA holder is adapted to the FLEX EA series, it can easily be adapted for other lateral wall EAs.

The synchronous video data was helpful in interpreting the recorded experimental data and is therefore recommended for intraoperative use. Force peaks could often be attributed to events such as bone contact or EA buckling. This is especially important given the limitation posed by possible bone contact. As the experimental data illustrated the large noise caused by contact forces, this has to be addressed in surgeon training, so that if possible, the insertion can be performed without any bone contact. Ultimately however, the prioritization between data quality and an optimal insertion for the patient must be made by the surgeon, should the two be mutually exclusive.

Forces and orientations were reliably measured without any errors. There are some factors influencing the varying peak forces during the insertion. The tissue within the TB specimens is affected by the multiple insertions. Likewise, the EAs are pre-bent in different ways after each insertion. Even without these factors, surgeons would slightly vary the insertion each time. This is illustrated by the moderate correlation between the electrode insertion depth and the peak force, showing that forces are influenced by the individual decision to end the insertion. The lack of correlation to the angular insertion depth is unusual as this is the more common unit to measure insertion depth and multiple publications directly link it to the insertion forces [12, 18]. The singular nature of the peak force might have an influence on the strength of the correlation as the maximum fore during the whole insertion is not necessarily representative of the average forces at the final insertion depth. Independent of correlation strength, the focus of this study was not to obtain information on the EA or the TB through the forces but rather to explore the type and value of data that can be collected. The varying conditions were therefore likely beneficial to the study as they increased the variations in the recorded insertions and thereby the types of force profiles recorded.

**Fig. 6:**
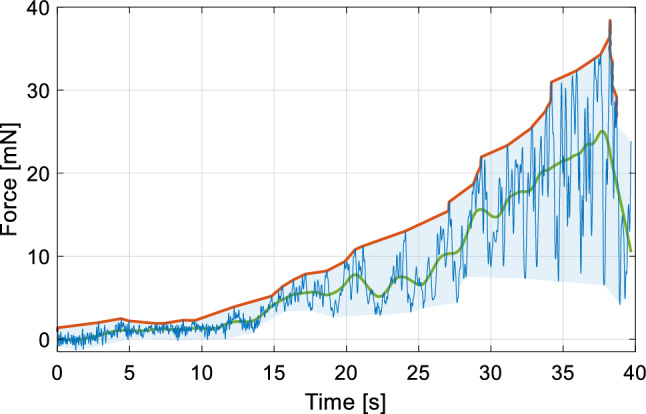
Representative example of the force profile (blue line) of an insertion trial with a moving average (green) and the upper border (red) of the convex hull outlined by the forces (light blue) highlighted

A typical force profile is shown in Fig. 6. In the beginning of the depicted insertion trial, there is little contact between the EA and the tissue. When the EA is advanced, the average forces and the amplitude of fluctuations in the force both increase. After the insertion, a brief decrease in forces is visible as the manual pressure is reduced. Naturally, the recorded forces differ from insertion forces recorded in a test bench, mostly due to human natural tremor. However, the typical rise in the forces toward the end of the insertion, resembling exponential growth was visible in the data [18, 19]. As due to the natural tremor, the forces acting on the EA are exerted in pulses, the peaks of these pulses are a good conservative estimate for forces transmitted to the cochlea. Considering a sliding average of the force would probably underestimate the transmitted forces.

A challenge in the analysis of the forces is the missing data on electrode insertion depth and speed. Typically, the force is analyzed relative to these factors, as this makes multiple insertions more comparable (e.g., by computing the insertion work). With only the timestamp of the force, information on the exact motion a surgeon performs can only be estimated. While the camera footage could be analyzed, this is only a rough estimate that would highly depend on the camera position with respect to the current trajectory. As data on the spatial motion of the tool would further improve data quality and interpretability, the integration of motion tracking is highly desirable for future research.

The tip fold-over detected in the fifth trial was only observed after the insertion on the CBCT images. As tip fold-overs are very rare in flexible lateral wall EAs [20], this was not an anticipated event. We believe this was connected to the changing tissue properties of the specimens due to the repeated insertions. The measured forces are distorted by some bone contact during the trial and the fact that during the bone contact the surgeon contacted the EA between the tool and the cochlea. While there is a force peak during this period, it is unclear whether it is caused by the tip fold-over or by the contact forces as the insertion depth continues to increase. As these are rare events, the extent of force associated with them is unknown, therefore it is not possible to assess whether the tool could have identified or even predicted this event. A study where such events are provoked could gather valuable data on predictable events.

When interpreting the measured forces, a major factor that needs to be taken into account are the extracochlear friction forces. While the aim is to measure intracochlear forces, the EA inevitably touches sections of the bone and slides along the RW. These forces are measured by the tool without a doubt and we assume that they are constant while the forces originating in the gradually increased electrode insertion depth are increasing as the insertion progresses. This is supported by the fact that there is a correlation between electrode insertion depth and insertion force maxima, as this would not be caused by extracochlear effects. The forces provided by our tool are not directly comparable to laboratory results due to the presence of the RW and the fact that laboratory conditions always include deviations from reality. However, we conclude that the tool is able to provide data approximating true intracochlear forces.

## Conclusion

The results from the evaluation experiments show that the insertion force measurement tool performs well in a realistic environment and that meaningful data can be obtained. Surgeons evaluated the surgical workflow with the tool to be equivalent to standard surgery in terms of handling, workflow, visibility and success of the insertion. Typical challenges faced in handling the tool were recorded and can easily be avoided through targeted surgeon training. The experimental data will facilitate the analysis of in vivo measurements. Based on this evaluation, the tool is ready for intraoperative use.

The ability to measure the spatial position of the tool would be a valuable upgrade. This would enable the analysis of electrode insertion depth and speed. In a more advanced setting, the tool could be registered to the patient and the surgeon could obtain information on an optimal insertion trajectory along which forces are likely minimal.

While the current version of the tool only records data for analysis after the insertion, real-time force feedback to the surgeon would be an important addition. There are many modalities available for such feedback and their efficacy would need to be evaluated. Live feedback can warn surgeons of force peaks below their perception and help to further minimize insertion forces.

### Supplementary Information

Below is the link to the electronic supplementary material.

Supplementary file1 (MP4 23207 KB)
